# Perioperative Serum Albumin and White Blood Cell Count for Early Postoperative Risk Stratification of Superficial Surgical Site Infection After Colorectal Cancer Surgery

**DOI:** 10.3390/cancers18162596

**Published:** 2026-08-12

**Authors:** Chihiro Kosugi, Kiyohiko Shuto, Mikito Mori, Daisuke Suzuki, Akihiro Usui, Yoshito Oka, Hiroaki Shimizu

**Affiliations:** Department of Surgery, Teikyo University Chiba Medical Center, Ichihara 299-0111, Chiba, Japan; shutou.kiyohiko.vb@teikyo-u.ac.jp (K.S.); mikimori45@yahoo.co.jp (M.M.); suzuki.daisuke.kz@teikyo-u.ac.jp (D.S.); usui.akihiro.gj@teikyo-u.ac.jp (A.U.); oka.yoshito.st@teikyo-u.ac.jp (Y.O.); shimizu.hiroaki.gk@teikyo-u.ac.jp (H.S.)

**Keywords:** colorectal cancer, surgical site infection, inflammation-based biomarker, nutritional biomarker, albumin, white blood cell count

## Abstract

**Background:** Surgical site infection (SSI) is a common complication after colorectal cancer (CRC) surgery. We evaluated routinely available perioperative biomarkers for early prediction of superficial SSI after curative CRC surgery. **Methods:** This retrospective study included 488 patients undergoing elective curative CRC resection. Preoperative and postoperative day (POD) 1 laboratory parameters were evaluated using receiver operating characteristic curve analysis and multivariable logistic regression. **Results:** Superficial SSI developed in 66 patients (13.5%). In the perioperative multivariable association model, preoperative serum albumin ≤ 3.70 g/dL (odds ratio [OR], 1.918; 95% confidence interval [CI], 1.066–3.449; *p* = 0.030) and WBC count ≥ 6.30 × 10^3^/µL (OR, 1.970; 95% CI, 1.123–3.458; *p* = 0.018) were independently associated with superficial SSI. On POD1, serum albumin ≤ 2.80 g/dL (OR, 3.447; 95% CI, 1.993–5.963; *p* < 0.001) and WBC count ≥ 10.50 × 10^3^/µL (OR, 2.822; 95% CI, 1.600–4.976; *p* < 0.001) remained independently associated with SSI. Although lower POD1 lymphocyte-to-monocyte ratio was associated with SSI in univariable analysis, it was not significant after adjustment. A combined model incorporating dichotomized preoperative and POD1 albumin and WBC counts showed modest discrimination (AUC, 0.731; 95% CI, 0.663–0.800), with 47.0% sensitivity and 89.3% specificity. **Conclusions:** Perioperative albumin and WBC counts were independently associated with superficial SSI. POD1 assessment may aid early risk stratification; however, the modest performance indicates that these biomarkers should complement rather than replace clinical surveillance. Prospective external validation is required.

## Simple Summary

Surgical site infection is a common complication after colorectal cancer surgery and can adversely affect postoperative recovery. This study examined whether routinely measured blood markers before and shortly after surgery could help identify patients at increased risk of superficial surgical site infection. Among 488 patients undergoing curative colorectal cancer surgery, 66 (13.5%) developed superficial surgical site infection. Lower preoperative serum albumin and an elevated white blood cell count were independently associated with infection. On postoperative day 1, lower serum albumin and an elevated white blood cell count also remained independently associated with infection, whereas the lymphocyte-to-monocyte ratio was not an independent factor. A combined model incorporating preoperative and postoperative day 1 albumin and white blood cell counts showed modest discriminatory ability. These routinely available biomarkers may complement clinical surveillance but are not sufficiently accurate for use as a stand-alone screening tool.

## 1. Introduction

Colorectal cancer (CRC) is one of the most common malignancies worldwide and is the most frequently diagnosed cancer in Japan, affecting more than 150,000 individuals annually [1]. Despite substantial advances in systemic chemotherapy, radiotherapy, and perioperative management, curative surgical resection remains the cornerstone of treatment for patients with localized CRC. As the number of patients undergoing colorectal resection continues to increase, minimizing postoperative complications has become an essential component of perioperative care.

Among postoperative complications, surgical site infection (SSI) remains one of the most frequent and clinically important adverse events after colorectal surgery. The incidence of SSI following colorectal resection remains considerably higher than that reported after other gastrointestinal procedures, ranging from approximately 10% to 30%. In addition to increasing postoperative morbidity, prolonging hospitalization, and escalating healthcare costs, SSIs may adversely affect long-term oncologic outcomes by delaying adjuvant therapy, increasing the risk of tumor recurrence, and reducing survival [2,3,4,5,6,7]. According to the Japanese Society for Cancer of the Colon and Rectum (JSCCR), recurrence rates after curative resection are 3.7%, 13.3%, and 30.8% for stage I, II, and III disease, respectively [8]. These observations underscore the importance of preventing SSI to optimize both short- and long-term outcomes following colorectal cancer surgery.

Numerous patient- and procedure-related risk factors for SSI have been identified, including advanced age, obesity, diabetes mellitus, smoking, hypoalbuminemia, higher American Society of Anesthesiologists physical status (ASA-PS) classification, prolonged operative time, excessive intraoperative blood loss, blood transfusion, surgical approach, and previous abdominal surgery [9,10]. Although these factors are useful for identifying patients at increased baseline risk, many are either nonmodifiable or become clinically relevant only after surgery. Consequently, they provide limited information for identifying patients who are likely to develop SSI during the early postoperative period, when timely intervention may be most effective. Because routine laboratory testing is universally performed on postoperative day (POD) 1 after colorectal surgery, biomarkers obtained at this time point may offer a practical opportunity for early postoperative risk stratification before clinical manifestations of SSI become apparent.

Systemic inflammation plays a pivotal role in both tumor progression and the host response to surgical stress and infection. Routine laboratory markers, including the white blood cell count, neutrophil count, lymphocyte count, C-reactive protein (CRP) level, and procalcitonin level, are widely used to evaluate perioperative inflammatory status. More recently, composite inflammation-based indices derived from routine blood tests, such as the neutrophil-to-lymphocyte ratio (NLR), lymphocyte-to-monocyte ratio (LMR), platelet-to-lymphocyte ratio (PLR), prognostic nutritional index (PNI), systemic immune-inflammation index (SII), and systemic inflammation score (SIS), have attracted considerable attention as prognostic biomarkers in patients with CRC [6,11]. These indices reflect the balance between systemic inflammation, nutritional status, and host immune competence and therefore may also provide clinically relevant information regarding susceptibility to postoperative infectious complications. However, although the prognostic value of these biomarkers has been extensively investigated, their utility for predicting postoperative superficial SSI remains poorly defined.

In particular, few studies have comprehensively compared the predictive value of preoperative inflammatory and nutritional biomarkers with that of biomarkers measured immediately after surgery for the early identification of patients at increased risk of superficial SSI. Clarifying whether routinely available POD1 laboratory parameters can identify high-risk patients before the onset of clinical signs of infection could facilitate intensified postoperative surveillance and targeted preventive strategies.

Therefore, the present study aimed to evaluate the predictive value of routinely available preoperative and postoperative inflammatory and nutritional biomarkers for superficial SSI after curative colorectal cancer surgery, with particular emphasis on POD1 biomarkers as practical tools for early postoperative risk stratification. POD1 was selected because it represents the earliest postoperative time point at which routine laboratory data are consistently available in our perioperative practice, thereby providing an opportunity for risk assessment before the clinical manifestation of SSI. We did not assume that POD1 represents the optimal postoperative time point for biomarker-based prediction.

## 2. Patients and Methods

### 2.1. Patients

This retrospective observational study included consecutive patients who underwent elective curative resection for histologically confirmed primary CRC at Teikyo University Chiba Medical Center between August 2013 and March 2017. During the study period, 560 patients underwent surgery for primary CRC and were screened for eligibility.

Patients were eligible for inclusion if they underwent elective curative resection for primary CRC. Exclusion criteria were an abdominal incision length of <4 cm, emergency surgery for ongoing intra-abdominal infection (e.g., generalized peritonitis secondary to colorectal perforation), pre-existing abdominal wall infection, palliative stoma creation without primary tumor resection, and any condition that precluded complete skin closure at the end of the primary procedure.

### 2.2. Perioperative Protocol

All patients underwent a standardized perioperative management protocol. Mechanical bowel preparation was performed without oral antibiotic bowel preparation or enemas, and patients fasted from the day before surgery. Prophylactic antibiotics were administered from the preoperative period through POD 2. Flomoxef (Shionogi, Osaka, Japan) was administered intravenously at a dose of 1 g 30 min before skin incision, followed by 1 g every 3 h during surgery and an additional 1 g dose 3 h after surgery. Beginning on POD1, 1 g of flomoxef was administered every 12 h, and prophylactic antibiotic therapy was discontinued after POD2.

Following bowel resection, with or without colorectal anastomosis, the abdominal cavity was irrigated with warm sterile saline (approximately 2000–3000 mL for open surgery and 1000 mL for laparoscopic surgery). After fascial closure with absorbable sutures (1-0 VICRYL; Johnson & Johnson, Somerville, NJ, USA), the wound was routinely irrigated with warm sterile saline before skin closure. Skin closure was performed using either 3-0 nylon sutures, skin staples, or subcuticular closure with 3-0 VICRYL, at the discretion of the operating surgeon.

### 2.3. Definition of Superficial Surgical Site Infection

Superficial SSI was defined according to the Centers for Disease Control and Prevention (CDC) criteria as an infection involving only the skin and subcutaneous tissue occurring within 30 postoperative days. Surgical wounds were routinely assessed at least twice daily during hospitalization by the attending surgeons and surgical staff using the CDC diagnostic criteria. All patients were additionally evaluated at hospital discharge and during routine outpatient follow-up for 30 days after surgery. When superficial SSI was clinically suspected, wound cultures were obtained at the discretion of the treating surgeon.

### 2.4. Ethics

The study protocol was approved by the Ethics Committee of Teikyo University Chiba Medical Center (No. 18-171-2). Written informed consent was obtained from all patients before surgery. The study was conducted in accordance with the Declaration of Helsinki and the Japanese Ethical Guidelines for Clinical Research.

### 2.5. Peripheral Blood Tests and Inflammatory Indices

Peripheral blood samples were collected within 1 week before surgery and during routine morning blood sampling on POD 1. Routine laboratory measurements included the white blood cell (WBC) count, neutrophil count, lymphocyte count, monocyte count, hemoglobin level, platelet count, and serum albumin level.

The following inflammation-based indices were calculated using both the preoperative and POD1 laboratory data:

• Neutrophil-to-lymphocyte ratio (NLR) = neutrophil count/lymphocyte count;
• Lymphocyte-to-monocyte ratio (LMR) = lymphocyte count/monocyte count;
• Platelet-to-lymphocyte ratio (PLR) = platelet count/lymphocyte count;
• Prognostic nutritional index (PNI) = 10 × serum albumin (g/dL) + 0.005 × peripheral lymphocyte count (/mm^3^);
• Systemic immune-inflammation index (SII) = platelet count × neutrophil count/lymphocyte count.

The SIS was calculated according to the original method described by Suzuki et al. [12]. Patients with both a serum albumin level > 4.0 g/dL and an LMR > 4.44 were assigned a score of 0, those with either a serum albumin level ≤ 4.0 g/dL or an LMR ≤ 4.44 were assigned a score of 1, and those with both a serum albumin level ≤ 4.0 g/dL and an LMR ≤ 4.44 were assigned a score of 2.

### 2.6. Statistical Analyses

Categorical variables were compared using Fisher’s exact test for 2 × 2 tables or the chi-square test for variables with more than two categories, as appropriate. Continuous variables were compared using the Mann–Whitney U test. Descriptive values are presented as mean ± standard deviation, except for age, which is presented as median and range.

Receiver operating characteristic (ROC) curve analysis was performed to evaluate the ability of preoperative and POD1 laboratory variables to discriminate patients who developed superficial SSI. AUCs and 95% CIs were calculated using DeLong’s method. Optimal laboratory cutoff values were determined by maximizing the Youden index (sensitivity + specificity − 1), with the direction of risk defined according to whether higher or lower values were associated with SSI. Continuous laboratory variables were subsequently dichotomized using these cutoffs for logistic regression. The preoperative SIS was categorized according to the original criteria proposed by Suzuki et al. [12]. POD1 SIS was not analyzed because the original score was developed for preoperative assessment and showed a marked postoperative ceiling effect. Age, BMI, operative time, and intraoperative blood loss were dichotomized using their cohort median values (69 years, 22.7 kg/m^2^, 160 min, and 54 mL, respectively).

Univariable logistic regression analysis was performed to examine the association between each clinical, operative, and laboratory variable and superficial SSI. Candidate variables for multivariable analysis were selected on the basis of their univariable associations, clinical relevance, and potential redundancy with other laboratory indices. Because several inflammation-based indices are mathematically derived from the same blood cell components and are therefore highly correlated, closely related variables were not simultaneously entered into the same multivariable model. Multicollinearity was assessed using variance inflation factors (VIFs).

For the perioperative multivariable association model, preoperative serum albumin, preoperative WBC count, preoperative LMR, pathological stage, intraoperative blood loss, and surgical approach were included to represent nutritional status, systemic inflammation, host immune-inflammatory status, tumor burden, and operative factors. This model was intended to estimate adjusted perioperative associations rather than provide strictly preoperative risk prediction. For the POD1 multivariable model, serum albumin, WBC count, and LMR were included as representative nutritional and inflammatory biomarkers while minimizing multicollinearity.

Odds ratios (ORs) and 95% confidence intervals (CIs) were calculated. A combined logistic regression model was constructed using the dichotomized preoperative and POD1 serum albumin and WBC variables. Its discriminatory performance was evaluated by ROC curve analysis, and the cutoff for the predicted probability was selected using the maximum Youden index.

All statistical analyses were performed using IBM SPSS Statistics version 30.0 (IBM Corp., Armonk, NY, USA). All tests were two-sided, and *p* < 0.05 was considered statistically significant.

## 3. Results

**Figure 1:**
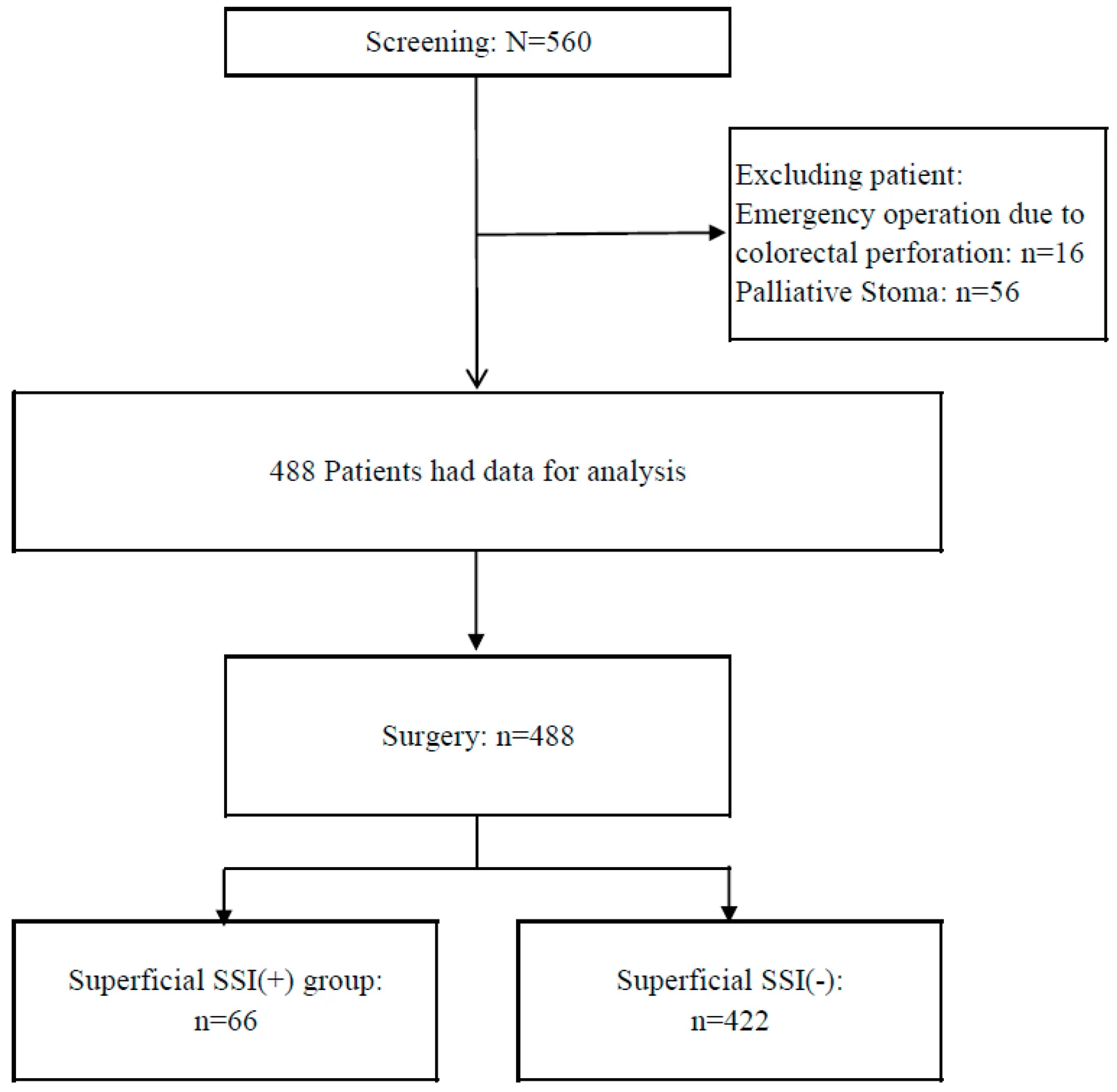
Flowchart showing a selection of patients on the basis of exclusion criteria.

Between August 2013 and March 2017, 560 patients with colorectal cancer were screened for eligibility. After application of the predefined exclusion criteria, 72 patients were excluded, including 16 who underwent emergency surgery for generalized peritonitis secondary to colorectal perforation and 56 who underwent stoma creation without primary tumor resection. Consequently, 488 patients were included in the final analysis (Figure 1). Of these, 66 patients (13.5%) developed superficial SSI within 30 postoperative days.

The baseline characteristics, preoperative laboratory findings, and intraoperative variables of patients with and without superficial SSI are summarized in Table 1. The groups were comparable with respect to age, sex, BMI, smoking status, diabetes mellitus, chronic kidney disease, hypertension and/or antihypertensive medication use, preoperative steroid use, and ASA-PS. Preoperative serum albumin levels were significantly lower in patients who subsequently developed superficial SSI (*p* < 0.001), and pathological stage IV disease was more frequent in the SSI group (*p* = 0.001). The skin-closure method also differed between the groups (*p* < 0.001).

With respect to preoperative inflammatory and nutritional markers, patients who developed superficial SSI had significantly higher WBC, neutrophil, monocyte, and platelet counts, NLR, and SII, whereas hemoglobin, LMR, and PNI were significantly lower. Preoperative PLR did not differ significantly when analyzed as a continuous variable. Operative time was longer (*p* = 0.016), intraoperative blood loss was greater (*p* = 0.002), and open surgery was more frequent (*p* < 0.001) in the superficial SSI group.

Postoperative day (POD) 1 laboratory findings are summarized in Table 2. Compared with patients who did not develop superficial SSI, those who subsequently developed SSI had significantly higher WBC and neutrophil counts, NLR, and SII, whereas serum albumin, PNI, and hemoglobin were significantly lower. Platelet count, PLR, LMR, lymphocyte count, and monocyte count did not differ significantly between the groups when analyzed as continuous variables.

### 3.1. Receiver Operating Characteristic Curve Analysis and Cutoff Values

Receiver operating characteristic (ROC) curve analysis was performed to evaluate the discriminatory performance of preoperative and POD 1 laboratory variables for predicting superficial SSI and to determine the optimal cutoff values using the maximum Youden index (Table 3).

Among the preoperative variables, serum albumin demonstrated the highest discriminatory performance (AUC, 0.667; 95% CI, 0.590–0.744), followed by PNI (AUC, 0.646; 95% CI, 0.570–0.721) and monocyte count (AUC, 0.634; 95% CI, 0.563–0.706). Among the POD1 variables, serum albumin showed the highest discriminatory performance (AUC, 0.699; 95% CI, 0.625–0.773), followed by PNI (AUC, 0.691; 95% CI, 0.618–0.764). POD1 LMR showed limited discrimination (AUC, 0.537; 95% CI, 0.452–0.622), with a cutoff of ≤1.61, sensitivity of 34.8%, and specificity of 78.9%.

The optimal laboratory cutoff values identified by ROC analysis were used for subsequent logistic regression analyses. Age, BMI, operative time, and intraoperative blood loss were dichotomized at their cohort medians. Preoperative SIS was analyzed according to the original criteria proposed by Suzuki et al. [12].

### 3.2. Univariable and Multivariable Logistic Regression Analyses

Univariable logistic regression analysis of clinical, operative, and clinicopathological variables demonstrated that preoperative hypoalbuminemia, greater intraoperative blood loss, open surgery, and pathological stage IV disease were significantly associated with superficial SSI (Table 4). Male sex showed a borderline association (*p* = 0.050).

Among the preoperative laboratory variables dichotomized using the recalculated ROC-derived cutoffs, low serum albumin, elevated WBC, neutrophil, monocyte, and platelet counts, elevated NLR, PLR, and SII, and low LMR, PNI, and hemoglobin were significantly associated with superficial SSI; lymphocyte count was not significant (Table 5). In the perioperative multivariable association model, preoperative serum albumin ≤ 3.70 g/dL (OR, 1.918; 95% CI, 1.066–3.449; *p* = 0.030) and WBC count > 6.30 × 10^3^/µL (OR, 1.970; 95% CI, 1.123–3.458; *p* = 0.018) remained independently associated with superficial SSI (Table 6). Pathological stage IV disease was not statistically significant after adjustment (OR, 1.843; 95% CI, 0.936–3.631; *p* = 0.077), and preoperative LMR was also not independently associated with SSI (OR, 1.432; 95% CI, 0.794–2.583; *p* = 0.233).

For the POD1 laboratory variables, hypoalbuminemia, elevated WBC and neutrophil counts, elevated platelet count, NLR, PLR, and SII, and low LMR, PNI, and hemoglobin were significantly associated with superficial SSI in univariable analyses (Table 7). In the multivariable model, POD1 serum albumin < 2.80 g/dL (OR, 3.447; 95% CI, 1.993–5.963; *p* < 0.001) and WBC count ≥ 10.50 × 10^3^/µL (OR, 2.822; 95% CI, 1.600–4.976; *p* < 0.001) remained independently associated with superficial SSI (Table 8). Although POD1 LMR ≤ 1.61 was associated with superficial SSI in univariable analysis (OR, 2.001; 95% CI, 1.146–3.496; *p* = 0.015), it was no longer significant after adjustment (OR, 1.467; 95% CI, 0.805–2.674; *p* = 0.210). VIF values were ≤1.75 in the perioperative model and ≤1.11 in the POD1 model, indicating no problematic multicollinearity.

**Figure 2:**
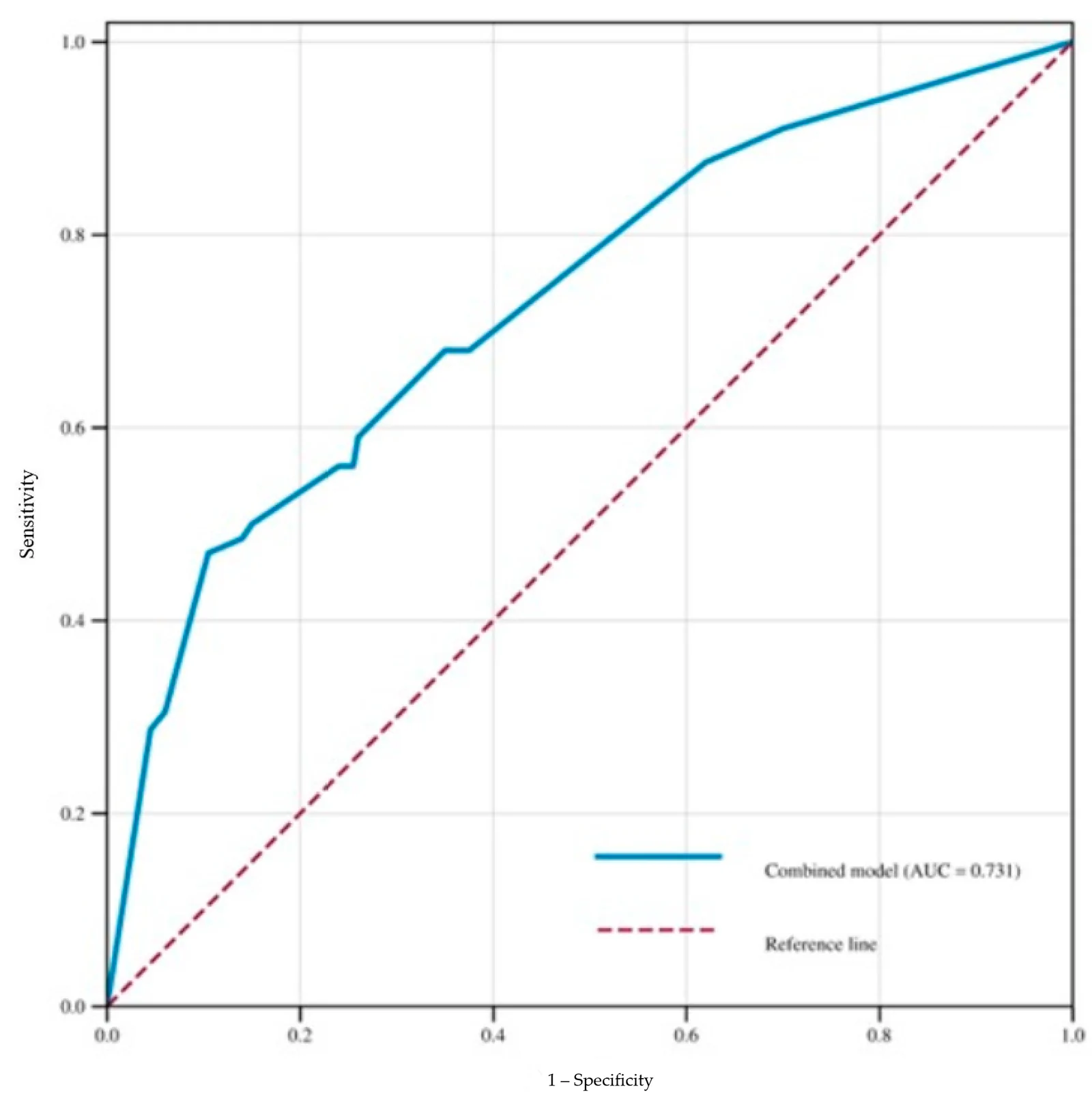
Receiver operating characteristic curve of the combined predictive model for superficial surgical site infection. The model incorporated preoperative serum albumin, preoperative white blood cell count, postoperative day 1 serum albumin, and postoperative day 1 white blood cell count. The area under the curve was 0.731 (95% confidence interval, 0.663–0.800), with a sensitivity of 47.0% and specificity of 89.3%.

Finally, a combined logistic regression model incorporating dichotomized preoperative and POD1 serum albumin and WBC variables demonstrated modest discriminatory ability for superficial SSI, with an AUC of 0.731 (95% CI, 0.663–0.800), sensitivity of 47.0%, and specificity of 89.3% at the maximum Youden index (Figure 2).

## 4. Discussion

In this retrospective study of 488 patients undergoing colorectal resection for CRC, lower preoperative serum albumin and an elevated preoperative WBC count were independently associated with superficial SSI in an adjusted perioperative association model. On POD1, lower serum albumin and an elevated WBC count remained independently associated with subsequent SSI. Although lower POD1 LMR was associated with SSI in univariable analysis, it was no longer significant after adjustment. A combined model incorporating dichotomized preoperative and POD1 serum albumin and WBC variables demonstrated modest discrimination (AUC = 0.731; 95% CI = 0.663–0.800), with sensitivity of 47.0% and specificity of 89.3%. These results suggest that perioperative hypoalbuminemia and systemic inflammatory responses may provide complementary risk information, but the model is insufficient for stand-alone screening.

Low preoperative serum albumin was independently associated with an increased risk of superficial SSI, and postoperative hypoalbuminemia on POD1 was also associated with subsequent SSI development. Serum albumin is widely recognized as a marker of nutritional status and systemic inflammation, both of which influence wound healing, immune function, and resistance to infection. Although the PNI, which incorporates serum albumin and lymphocyte count, was not identified as an independent predictor in multivariable analysis, our findings suggest that optimization of perioperative nutritional status may contribute to reducing the risk of postoperative SSI. Moreover, the strong correlation between serum albumin and PNI supports the use of serum albumin as a more direct and readily interpretable representative marker of nutritional and inflammatory status in the multivariable model.

WBC count was the other biomarker that remained independently associated with superficial SSI at both the preoperative and POD1 time points. An elevated preoperative WBC count may reflect underlying systemic inflammatory activation, whereas an elevated POD1 WBC count may additionally reflect the magnitude of the early inflammatory response to surgical stress. Although neutrophil count, NLR, PLR, and SII were also associated with SSI in univariate analyses, these indices share substantial biological and mathematical overlap with WBC and its cellular components. To reduce multicollinearity and model complexity in the setting of a limited number of SSI events, WBC count was selected as a representative marker of systemic inflammation. Its routine availability, simplicity, and relatively favorable discriminatory performance on POD1 (AUC = 0.661) also support its potential usefulness for early postoperative risk assessment. Nevertheless, postoperative leukocytosis is nonspecific and may reflect surgical stress rather than impending infection; therefore, WBC count should not be interpreted in isolation.

In addition to nutritional status, we comprehensively evaluated inflammation-based biomarkers. These biomarkers have been widely investigated as prognostic indicators in patients with malignancies. Among them, the NLR, PLR, and LMR reflect the balance between systemic inflammation and host immune competence. Previous studies have demonstrated that elevated NLR and reduced LMR are associated with unfavorable oncologic outcomes in CRC and other solid tumors [13,14,15,16]. Chronic systemic inflammation also contributes to colorectal carcinogenesis, tumor progression, angiogenesis, and metastatic dissemination [17,18,19,20]. Furthermore, Suzuki et al. reported that the SIS, which combines serum albumin and LMR, is strongly associated with long-term prognosis in patients with CRC [12]. Collectively, these findings support the hypothesis that perioperative nutritional and inflammatory status may also influence susceptibility to postoperative infectious complications.

Particular attention should be paid to the findings regarding LMR. Lymphocytes play a central role in cell-mediated immunity and host defense against infection, whereas monocytes participate in innate immune and inflammatory responses [21,22,23]. In the present study, a POD1 LMR ≤ 1.61 was associated with superficial SSI in univariable analysis. However, LMR alone demonstrated little discriminatory ability (AUC = 0.537; 95% CI = 0.452–0.622), and its association was no longer significant after adjustment for POD1 serum albumin and WBC count (adjusted OR = 1.467; 95% CI = 0.805–2.674; *p* = 0.210). Thus, the present findings do not support POD1 LMR as an independent predictor or effective stand-alone discriminator of superficial SSI. The original SIS was retained only for preoperative assessment because applying it on POD1 produced a pronounced ceiling effect caused by postoperative hypoalbuminemia and an apparently reversed association that lacked clinical interpretability.

Pathological stage was associated with superficial SSI in univariable analysis, with stage IV disease being more frequent among patients who developed SSI. This association was attenuated after multivariable adjustment (adjusted OR = 1.843; 95% CI = 0.936–3.631; *p* = 0.077). Patients with advanced disease may have poorer nutritional status, greater systemic inflammatory activity, immunological impairment, and more complex operative requirements. However, this model was an adjusted perioperative association model rather than a strictly preoperative prediction model, and the observational findings should not be interpreted causally.

Surgical approach was another important factor in the univariate analysis. Laparoscopic surgery was associated with a substantially lower unadjusted risk of superficial SSI than open surgery, consistent with the potential wound-related advantages of minimally invasive surgery [9,10]. However, this association was attenuated after adjustment and was no longer statistically significant. This attenuation may reflect differences in patient selection, disease stage, operative complexity, and other perioperative characteristics between laparoscopic and open procedures. Accordingly, the present observational data do not permit a causal conclusion regarding surgical approach and SSI. Similarly, although potential differences in biomarker performance between colon and rectal resections or between laparoscopic and open procedures are clinically relevant, the relatively small number of SSI events limited adequately powered subgroup analyses. Larger studies are required to determine whether the predictive value of these biomarkers varies according to tumor location or surgical approach.

An important consideration is the timing of postoperative biomarker assessment. POD1 was selected in the present study primarily because our objective was to identify patients at increased risk of superficial SSI as early as practically possible after surgery. Routine blood sampling is commonly performed on POD1 after colorectal surgery, making this time point readily available without requiring additional blood collection. Importantly, in our cohort, differences in several inflammatory and nutritional biomarkers between patients with and without subsequent SSI were already evident on POD1, suggesting that clinically relevant host responses may be detectable at this early stage. Thus, POD1 was selected as a pragmatic time point for early postoperative risk stratification rather than as a priori evidence that it represents the biologically optimal time point.

Later postoperative measurements, particularly on POD2 and POD3, may provide additional or complementary predictive information because inflammatory and nutritional biomarkers undergo dynamic changes following surgical stress. Previous studies of colorectal surgery have demonstrated the clinical utility of later postoperative inflammatory markers, particularly C-reactive protein (CRP). In patients undergoing curative colorectal cancer surgery, POD2 CRP was associated with subsequent POD3 and POD4 CRP levels and with thresholds previously associated with postoperative infective complications [24]. Similarly, POD3 CRP demonstrated good discriminatory ability for postoperative infective complications after colorectal cancer surgery, with an area under the ROC curve of 0.80 [25]. A diagnostic meta-analysis including 1832 patients further reported that CRP had its best overall predictive performance on POD4, with a pooled AUC of 0.81 for postoperative infectious complications [26]. These findings suggest that later postoperative measurements may provide stronger discrimination for established postoperative inflammatory or infectious complications than very early measurements. However, later measurements may also provide a narrower window for early risk stratification and preventive intervention.

The temporal behavior of serum albumin also supports the importance of considering the timing of assessment. In a study of 626 patients undergoing colorectal surgery, a reduction in serum albumin within the first 2 postoperative days was independently associated with postoperative complications, and patients with a reduction of ≥15% experienced a higher incidence of surgical site infections [27]. Thus, postoperative nutritional and inflammatory biomarkers may provide clinically meaningful information at different stages of the postoperative course. Nevertheless, the primary aim of the present study was not to determine the single optimal postoperative time point, but rather to evaluate whether routinely available POD1 biomarkers could facilitate early postoperative risk stratification before overt clinical manifestations of SSI became apparent.

The combined model incorporating dichotomized preoperative and POD1 serum albumin and WBC variables achieved an AUC of 0.731 (95% CI = 0.663–0.800), compared with an AUC of 0.699 (95% CI = 0.625–0.773) for POD1 serum albumin alone. These confidence intervals overlapped substantially. Because a formal paired comparison of AUCs using the DeLong test was not performed, the numerical difference should not be interpreted as evidence of a statistically significant improvement. At the maximum Youden index, the combined model had sensitivity of 47.0% and specificity of 89.3%, indicating that more than half of patients who subsequently developed superficial SSI would not be identified. Accordingly, the model should not be used to rule out SSI or replace routine postoperative clinical surveillance. Its incremental value remains modest and requires prospective external validation.

Notably, the present analysis focused on routinely available POD1 laboratory parameters and did not incorporate CRP into the prediction model. CRP is a well-established postoperative inflammatory marker, particularly when measured on POD3 or POD4 [24,25,26,27,28,29]. Therefore, the present results should not be interpreted as demonstrating superiority of serum albumin or WBC count over CRP. Rather, they indicate that clinically relevant differences in nutritional and inflammatory status can already be detected using routine laboratory measurements on POD1. Future studies directly comparing POD1 biomarkers with serial CRP measurements will be needed to determine whether these parameters provide incremental information beyond established postoperative inflammatory markers.

The potential clinical application of these findings should also be considered cautiously. Identification of patients with perioperative hypoalbuminemia and an elevated POD1 WBC count could prompt more frequent wound assessment, closer monitoring for early local or systemic signs of infection, and earlier clinical reassessment when abnormalities develop. Our previous randomized controlled trial demonstrated that silver-containing Aquacel^®^ Ag Hydrofiber dressing (ConvaTec, Princeton, NJ, USA) significantly reduced the incidence of superficial SSI after lower gastrointestinal surgery [30]. Accordingly, enhanced wound protection or prolonged use of protective dressings could be investigated prospectively as a risk-adapted strategy in patients identified as being at increased risk. However, the present observational study did not evaluate biomarker-guided interventions, and it cannot establish that modifying postoperative management according to these biomarkers reduces SSI. Such strategies therefore require prospective interventional validation before routine implementation.

Strengths of the present study include the relatively large cohort of patients undergoing curative CRC resection and the use of a standardized perioperative management protocol within a single institution. These factors minimized variability in surgical technique and perioperative care, thereby allowing a more consistent evaluation of perioperative inflammatory biomarkers. Furthermore, unlike previous studies that focused primarily on preoperative inflammatory status or overall postoperative complications, the present study evaluated both preoperative and early postoperative biomarkers specifically in relation to superficial SSI, providing insight into dynamic perioperative changes in nutritional and immune status.

Several limitations should be acknowledged. First, this study was retrospective and conducted at a single institution in Japan. In addition, only 66 patients developed superficial SSI, limiting the number of variables that could be reliably included in multivariable models and increasing the risk of model overfitting. Consequently, selection bias, residual confounding, and limited generalizability cannot be excluded. The exclusion of patients undergoing emergency surgery for active intra-abdominal infection, palliative stoma creation without tumor resection, and other procedures not meeting the predefined eligibility criteria resulted in a relatively homogeneous cohort but may further limit the applicability of the findings to emergency, palliative, or other higher-risk surgical populations. Second, highly correlated or mathematically overlapping biomarkers were not entered simultaneously into the multivariable models, and representative variables were selected to reduce model complexity and multicollinearity. Although this approach was intended to improve model stability given the limited number of SSI events, variable selection may nevertheless have influenced the resulting effect estimates. Third, continuous biomarkers were dichotomized using ROC-derived cutoff values obtained from the same cohort. Although this facilitates clinical interpretation, dichotomization results in loss of information and may exaggerate apparent associations, while deriving and evaluating cutoffs within the same dataset may lead to optimistic estimates of predictive performance. The reported cutoff values should therefore be considered exploratory and require independent validation. Fourth, no internal validation using bootstrapping or cross-validation and no external validation were performed. Therefore, the reported AUC and effect estimates may overestimate model performance in new patient populations. Fifth, inflammatory and nutritional biomarkers were assessed only before surgery and on POD1. Although POD1 was selected to enable early postoperative risk assessment, the present study cannot determine whether POD1 represents the optimal timing for SSI prediction. Serial measurements on POD2, POD3, or later may provide additional or superior predictive information. Finally, CRP was not included in the present analysis, preventing direct comparison of the proposed biomarkers with an established postoperative inflammatory marker.

## 5. Conclusions

In conclusion, lower preoperative and POD1 serum albumin levels and elevated WBC counts were independently associated with superficial SSI following curative colorectal cancer surgery. A lower POD1 LMR was associated with superficial SSI in univariable analysis but was not independently associated after adjustment. A combined model incorporating dichotomized preoperative and POD1 serum albumin and WBC variables showed modest discrimination (AUC, 0.731; sensitivity, 47.0%; specificity, 89.3%) and is insufficient as a stand-alone predictive tool. These routinely available biomarkers may complement, rather than replace, standard clinical surveillance. Prospective multicenter studies incorporating serial postoperative measurements and external validation are warranted.

## Figures and Tables

**Table 1:** Baseline characteristics of the included patients according to superficial surgical site infection status.

		Superficial SSI (+): n = 66		Superficial SSI (−): n = 422		*p*-Value
Age (years) ^a^		69	(42–89)	69	(29–90)	0.766
Sex (male:female)		50:16		267:155		0.052
Risk factors and comorbidities						
	BMI (kg/m^2^) ^b^	22.5 ± 3.1		22.8 ± 3.8		0.736
	Smoking	23	(34.8%)	125	(29.6%)	0.391
	Diabetes mellitus	20	(30.3%)	112	(26.5%)	0.552
	Chronic kidney disease (eGFR < 30 mL/min/1.73 m^2^)	5	(7.6%)	16	(3.8%)	0.184
	Arterial hypertension and/or antihypertensive medication	29	(43.9%)	204	(48.3%)	0.596
	Preoperative steroid medication	2	(3.0%)	13	(3.1%)	1.000
	ASA-PS (1–2:3–4)	48:18		338:84		0.192
	Preoperative serum albumin (g/dL) ^b^	3.5 ± 0.7		3.9 ± 0.5		<0.001
Location						
	Colon	36	(54.5%)	224	(53.1%)	0.895
	Rectum	30	(45.5%)	198	(46.9%)	
ypStage						
	0–III	49	(74.2%)	378	(89.6%)	0.001
	IV	17	(25.8%)	44	(10.4%)	
Preoperative blood parameter						
	White blood cell count (×10^3^/μL) ^b^	7.0 ± 2.4		6.0 ± 1.9		<0.001
	Neutrophil (×10^3^/μL) ^b^	4.6 ± 2.5		3.7 ± 1.6		<0.001
	Lymphocyte (×10^3^/μL) ^b^	1.7 ± 0.6		1.7 ± 0.6		0.693
	Monocyte (×10^2^/μL) ^b^	4.6 ± 1.8		3.9 ± 1.5		<0.001
	Platelet (×10^5^/μL) ^b^	27.5 ± 9.1		24.6 ± 8.1		0.006
	NLR ^b^	3.3 ± 2.8		2.6 ± 2.0		0.039
	PLR ^b^	183.6 ± 94.2		167.3 ± 91.9		0.135
	LMR ^b^	4.2 ± 2.2		4.8 ± 2.2		0.007
	PNI ^b^	43.9 ± 8.1		47.5 ± 6.8		<0.001
	SII ^b^	956.2 ± 960.3		653.4 ± 668.3		0.003
	SIS (0–1:2)	30:36		297:125		<0.001
	Hemoglobin (g/dL) ^b^	11.8 ± 2.0		12.4 ± 2.0		0.033
Surgeon grade						
	Consultant	44	(66.7%)	291	(68.9%)	0.902
	Senior training registrar	18	(27.3%)	110	(26.1%)	
	Junior training registrar	4	(6.0%)	21	(5.0%)	
Skin-closure method						
	Skin clips	54	(81.8%)	389	(92.2%)	<0.001
	Interrupted suture	10	(15.2%)	15	(3.6%)	
	Subcuticular suture	2	(3.0%)	18	(4.2%)	
Operative time (min) ^b^		229.7 ± 144.7		186.1 ± 110.6		0.016
Blood loss (mL) ^b^		859.7 ± 1751.5		236.5 ± 650.3		0.002
Laparoscopic surgery:open abdominal surgery		22:44		269:153		<0.001

^a^ Median (range); ^b^ mean ± standard deviation; SSI: surgical site infection; BMI: body mass index; eGFR: estimated glomerular filtration rate; ASA-PS: American Society of Anesthesiologists physical status; NLR: neutrophil-to-lymphocyte ratio; PLR: platelet-to-lymphocyte ratio; LMR: lymphocyte-to-monocyte ratio PNI: prognostic nutritional index; SII: systemic immune-inflammation index: SIS: systemic inflammation score.

**Table 2:** Blood parameter of postoperative day 1 of patients.

		Superficial SSI (+): n = 66	Superficial SSI (−): n = 422	*p*-Value
Blood parameter of postoperative day 1				
	Serum albumin (g/dL) ^a^	2.8 ± 0.5	3.1 ± 0.4	<0.001
	White blood cell count (×10^3^/μL) ^a^	11.7 ± 3.9	9.6 ± 3.2	<0.001
	Neutrophil (×10^3^/μL) ^a^	10.0 ± 4.1	8.0 ± 3.1	<0.001
	Lymphocyte (×10^3^/μL) ^a^	1.0 ± 0.4	1.1 ± 0.4	0.519
	Monocyte (×10^2^/μL) ^a^	4.9 ± 2.5	4.6 ± 2.0	0.513
	Platelet (×10^5^/μL) ^a^	24.6 ± 22.5	20.7 ± 9.5	0.240
	NLR ^a^	12.1 ± 8.8	9.7 ± 11.6	0.005
	PLR ^a^	304.0 ± 420.1	231.7 ± 165.5	0.270
	LMR ^a^	2.6 ± 1.6	2.6 ± 1.2	0.339
	PNI ^a^	33.0 ± 5.8	36.4 ± 5.1	<0.001
	SII ^a^	3144.1 ± 4249.7	2015.3 ± 2582.3	0.003
	Hemoglobin (g/dL) ^a^	10.9 ± 1.6	11.4 ± 1.8	0.028

^a^ Mean ± standard deviation; SSI: surgical site infection; NLR: neutrophil-to-lymphocyte ratio; PLR: platelet-to-lymphocyte ratio; LMR: lymphocyte-to-monocyte ratio; PNI: prognostic nutritional index; SII: systemic immune-inflammation index; SIS: systemic inflammation score.

**Table 3:** Receiver operating characteristic (ROC) curve analysis of preoperative and postoperative day 1 laboratory variables for predicting superficial surgical site infection.

Variables		AUC	95% CI	Cutoff Value	Sensitivity (%)	Specificity (%)
Serum albumin (g/dL)						
	preoperative	0.667	0.590–0.744	3.70	59.1	68.0
	postoperative day 1	0.699	0.625–0.773	2.80	54.5	76.3
White blood cell count (×10^3^/μL)						
	preoperative	0.639	0.568–0.710	6.30	60.6	62.1
	POD1	0.661	0.584–0.738	10.50	62.1	68.0
Neutrophil count (×10^3^/μL)						
	preoperative	0.631	0.558–0.704	3.42	69.7	53.6
	POD1	0.654	0.577–0.732	8.65	62.1	65.6
Lymphocyte count (×10^3^/μL)						
	preoperative	0.515	0.442–0.588	1.31	78.8	31.5
	POD1	0.525	0.449–0.601	1.16	71.7	37.0
Monocyte count (×10^2^/μL)						
	preoperative	0.634	0.563–0.706	4.10	56.1	67.3
	POD1	0.525	0.445–0.605	5.35	39.4	70.6
Platelet (×10^5^/μL)						
	preoperative	0.604	0.527–0.682	25.80	59.1	63.5
	POD1	0.545	0.459–0.631	17.50	39.4	76.1
NLR						
	preoperative	0.579	0.501–0.656	2.00	66.7	48.8
	POD1	0.608	0.534–0.682	7.91	63.6	55.0
PLR						
	preoperative	0.557	0.480–0.634	214.40	31.8	79.9
	POD1	0.542	0.462–0.622	464.70	16.7	96.7
LMR						
	preoperative	0.603	0.530–0.677	3.56	51.5	70.6
	POD1	0.537	0.452–0.622	1.61	34.8	78.9
PNI						
	preoperative	0.646	0.569–0.722	44.40	54.5	70.9
	POD1	0.691	0.618–0.764	35.20	71.2	60.9
SII						
	preoperative	0.613	0.535–0.691	567.50	59.1	61.1
	POD1	0.612	0.534–0.691	1668.10	63.6	60.0
Hemoglobin (g/dL)						
	preoperative	0.582	0.506–0.657	13.20	78.8	39.1
	POD1	0.435	0.513–0.654	12.00	75.8	41.5

AUC: areas under the curve; CI: confidence interval; POD1: postoperative day 1; NLR: neutrophil-to-lymphocyte ratio; PLR: platelet-to-lymphocyte ratio; LMR: lymphocyte-to-monocyte ratio; PNI: prognostic nutritional index; SII: systemic immune-inflammation index.

**Table 4:** Univariate risk estimates of acquiring a postoperative superficial surgical site infection.

Variable (Risk Category vs. Reference)		Superficial SSI (+): n = 66	Superficial SSI (−): n = 422	Odds Ratio	(95% CI)	*p*-Value
Age (years) ^a^ >69 vs. ≤69		31 (47.0%)	203 (48.1%)	0.956	(0.568–1.607)	0.864
Sex: Male vs. female		50 (75.8%)	267 (63.3%)	1.814	(0.999–3.295)	0.050
*Risk factors and comorbidities*						
	BMI (kg/m^2^) ^a^ > 22.7 vs. ≤22.7	33 (50.0%)	211 (50.0%)	1.000	(0.595–1.680)	1.000
	Smoking: yes vs. no	23 (34.8%)	125 (29.6%)	1.271	(0.735–2.198)	0.391
	Diabetes mellitus: yes vs. no	20 (30.3%)	112 (26.5%)	1.203	(0.682–2.123)	0.523
	Chronic kidney disease (eGFR < 30 mL/min/1.73 m^2^): yes vs. no	5 (7.6%)	16 (3.8%)	2.080	(0.735–5.882)	0.167
	Arterial hypertension and/or antihypertensive medication: yes vs. no	29 (43.9%)	204 (48.3%)	0.838	(0.497–1.412)	0.506
	Preoperative steroid medication: yes vs. no	2 (3.0%)	13 (3.1%)	0.983	(0.217–4.459)	0.982
	ASA-PS 3–4 vs. 1–2	18 (27.3%)	84 (19.9%)	1.509	(0.835–2.728)	0.173
	Preoperative serum albumin ≤ 3.7 vs. >3.7 (g/dL) ^a^	39 (59.1%)	135 (32.0%)	3.071	(1.805–5.225)	<0.001
Surgery						
	Location: Rectum vs. colon	30 (45.5%)	198 (46.9%)	0.943	(0.560–1.587)	0.824
	Operative time ≥ 160 vs. <160 (min) ^a^	38 (57.6%)	207 (49.1%)	1.410	(0.835–2.381)	
	Blood loss ≥ 54 vs. <54 (mL) ^a^	49 (74.2%)	196 (46.4%)	3.324	(1.854–5.959)	<0.001
	Open surgery vs. laparoscopic surgery	44 (66.7%)	153 (36.3%)	3.516	(2.031–6.088)	<0.001
Pathological Stage:ypStage IV vs. 0–III		17 (25.8%)	44 (10.4%)	2.981	(1.581–5.618)	<0.001

^a^ ROC cutoffs; SSI: surgical site infection; BMI: body mass index; ASA-PS: American Society of Anesthesiologists physical status.

**Table 5:** Univariable analysis of preoperative laboratory variables.

			Superficial SSI (+): n = 66		Superficial SSI (−): n = 422		Odds Ratio	(95% CI)	*p*-Value
Preoperatively									
	Preoperative serum albumin (g/dL) ^a^								
		>3.7	39	(59.1%)	135	(32.0%)	3.071	(1.805–5.225)	<0.001
		≤3.7	27	(40.9%)	287	(68.0%)			
	White blood cell count (×10^3^/μL) ^a^								
		>6.30	40	(60.6%)	160	(37.9%)	2.519	(1.481–4.286)	<0.001
		≤6.30	26	(39.4%)	262	(62.1%)			
	Neutrophil (×10^3^/μL) ^a^								
		>3.41	46	(69.7%)	196	(46.4%)	2.652	(1.517–4.637)	<0.001
		≤3.41	20	(31.8%)	227	(53.8%)			
	Lymphocyte (×10^3^/μL) ^a^								
		>1.31	52	(78.8%)	289	(68.5%)	1.709	(0.915–3.193)	0.093
		≤1.31	14	(21.2%)	133	(31.5%)			
	Monocyte (×10^2^/μL) ^a^								
		>4.10	37	(56.1%)	138	(32.7%)	2.626	(1.550–4.447)	<0.001
		≤4.10	29	(43.9%)	284	(67.3%)			
	Platelet (×10^5^/μL) ^a^								
		>25.8	39	(59.1%)	154	(36.5%)	2.514	(1.481–4.267)	<0.001
		≤25.8	27	(40.9%)	268	(63.5%)			
	NLR ^a^								
		>2.00	22	(33.3%)	206	(48.8%)	1.907	(1.105–3.294)	0.020
		≤2.00	44	(66.7%)	216	(51.2%)			
	PLR ^a^								
		>214.6	45	(68.2%)	337	(79.9%)	1.850	(1.046–3.272)	0.034
		≤214.6	21	(31.8%)	85	(20.1%)			
	LMR ^a^								
		>3.56	32	(48.5%)	298	(70.6%)	2.553	(1.509–4.322)	<0.001
		≤3.56	34	(51.5%)	124	(29.4%)			
	PNI ^a^								
		>44.4	36	(54.5%)	123	(29.1%)	2.917	(1.720–4.946)	<0.001
		≤44.4	30	(45.5%)	299	(70.9%)			
	SII ^a^								
		>567.5	39	(59.1%)	164	(38.9%)	2.272	(1.340–3.854)	0.002
		≤567.5	26	(39.4%)	258	(61.1%)			
	SIS ^a^								
		0–1	30	(45.5%)	297	(70.4%)	2.851	(1.682–4.833)	<0.001
		2	36	(54.5%)	125	(29.6%)			
	Hemoglobin (g/dL) ^a^								
		>13.2	14	(21.2%)	170	(39.1%)	2.851	(1.281–4.440)	0.006
		≤13.2	52	(78.8%)	257	(60.9%)			

^a^ Median values were used for the ROC cut off; SSI: surgical site infection; NLR: neutrophil-to-lymphocyte ratio; PLR: platelet-to-lymphocyte ratio; LMR: lymphocyte-to-monocyte ratio; PNI: prognostic nutritional index SII: systemic immune-inflammation index: SIS: systemic inflammation score.

**Table 6:** Perioperative multivariable association model for superficial SSI.

			95% CI		
Variable		OR	Lower	Upper	*p*-Value
	Preoperative serum albumin (g/dL) (cut-off 3.7)	1.918	1.066	3.449	0.030
	White blood cell count (×10^3^/μL) (cut-off 6.30)	1.970	1.123	3.458	0.018
	Blood loss (ml) (cut-off 54)	1.500	0.701	3.208	0.296
	Laparoscopic surgery vs. open surgery	1.826	0.882	3.780	0.105
	ypStageIV	1.843	0.936	3.631	0.077
	Preoperative LMR (cut-off 3.56)	1.432	0.794	2.583	0.233

OR: odds ratio; CI: confidence interval; ASA-PS; American Society of Anesthesiologists physical status; LMR: lymphocyte-to-monocyte ratio; SIS: systemic inflammation score.

**Table 7:** Univariate risk estimates of acquiring a postoperative superficial surgical site infection of blood parameter.

			Superficial SSI (+): n = 66		Superficial SSI (−): n = 422		Odds Ratio	(95% CI)	*p*-Value
1 Day postoperatively									
	Postoperative day1 serum albumin (g/dL) ^a^								
		≥2.8	30	(45.5%)	322	(77.3%)	3.864	(2.265–6.591)	<0.001
		<2.8	36	(54.5%)	100	(23.7%)			
	White blood cell count (×10^3^/μL) ^a^								
		≥10.50	41	(62.1%)	135	(32.0%)	3.487	(2.036–5.970)	<0.001
		<10.50	25	(37.9%)	287	(68.0%)			
	Neutrophil (×10^3^/μL) ^a^								
		≥8.65	41	(62.1%)	145	(34.4%)	3.133	(1.832–5.357)	<0.001
		<8.65	25	(37.9%)	277	(65.6%)			
	Lymphocyte (×10^3^/μL) ^a^								
		≥1.16	19	(28.8%)	156	(37.0%)	1.451	(0.822–2.561)	0.199
		<1.16	47	(71.2%)	266	(63.0%)			
	Monocyte (×10^2^/μL) ^a^								
		≥5.35	26	(39.4%)	124	(29.4%)	1.562	(0.914–2.671)	0.103
		<5.35	40	(60.6%)	298	(70.6%)			
	Platelet (×10^5^/μL) ^a^								
		≥23.7	26	(39.4%)	101	(23.9%)	2.066	(1.201–3.552)	0.009
		<23.7	40	(60.6%)	321	(76.1%)			
	NLR ^a^								
		≥7.91	42	(63.6%)	190	(45.0%)	2.137	(1.249–3.656)	0.006
		<7.91	24	(36.4%)	232	(55.0%)			
	PLR ^a^								
		≥543.0	11	(16.7%)	14	(3.3%)	5.829	(2.520–13.479)	<0.001
		<543.0	55	(83.7%)	408	(96.7%)			
	LMR ^a^								
		≥1.61	43	(65.2%)	333	(78.9%)	2.001	(1.146–3.496)	0.015
		<1.61	23	(34.8%)	89	(21.1%)			
	PNI ^a^								
		≥35.2	23	(34.8%)	257	(60.9%)	3.853	(2.184–6.797)	<0.001
		<35.2	47	(71.2%)	165	(39.1%)			
	SII ^a^								
		≥1668.1	42	(63.6%)	169	(40.0%)	2.620	(1.530–4.487)	<0.001
		<1668.1	24	(36.4%)	253	(60.0%)			
	Hemoglobin (g/dL) ^a^								
		≥12	16	(24.2%)	175	(41.5%)	2.214	(1.221–4.016)	0.009
		<12	50	(75.8%)	247	(58.5%)			

^a^ ROC cut off; SSI: surgical site infection; NLR: neutrophil-to-lymphocyte ratio; PLR: platelet-to-lymphocyte ratio; LMR: lymphocyte-to-monocyte ratio; PNI: prognostic nutritional index; SII: systemic immune-inflammation index; SIS: systemic inflammation score.

**Table 8:** Multivariable analysis of postoperative day 1 variables.

			95% CI		
Variable		OR	Lower	Upper	*p*-Value
	Postoperative day 1 serum albumin (g/dL) (cut-off 2.8)	3.447	1.993	5.963	<0.001
	Postoperative day 1 white blood cell count (×10^3^/μL) (cut-off 10.5)	2.822	1.600	4.976	<0.001
	postoperative day 1 LMR (cut-off 1.61)	1.467	0.805	2.674	0.210

OR: odds ratio; CI: confidence interval; LMR: lymphocyte-to-monocyte ratio.

## Data Availability

The datasets generated and/or analyzed during the current study are available from the corresponding author on reasonable request.

## Abbreviations

The following abbreviations are used in this manuscript:

CRC	colorectal cancer
SSI	surgical site infection
JSCCR	Japanese Society for Cancer of the Colon and Rectum
ASA-PS	American Society of Anesthesiologist physical status
POD	postoperative day
CRP	C-reactive protein
NLR	neutrophil-to-lymphocyte ratio
LMR	lymphocyte-to-monocyte ratio
PLR	platelet-to-lymphocyte ratio
PNI	prognostic nutritional index
SII	systemic immune-inflammation index
SIS	systemic inflammation score
CDC	Centers for Disease Control and Prevention
WBC	white blood cell
ROC	receiver operating characteristic
OR	odds ratio
CI	confidence interval
AUC	areas under the curve
